# Determinants of caregiver burden of persons with disabilities in a rural district in Egypt

**DOI:** 10.1186/s12889-020-09266-4

**Published:** 2020-07-23

**Authors:** Eman Ramadan Ghazawy, Eman Sameh Mohammed, Eman Mohamed Mahfouz, Marwa Gamal Abdelrehim

**Affiliations:** Department of Public Health and Preventive Medicine, Faculty of Medicine, Minia University, Minia, Egypt

**Keywords:** Caregivers, Burden, Disabled, Exploratory factor analysis, Egypt

## Abstract

**Background:**

Family caregivers are critical partners in the plan of care of people with disabilities. The study aims to demonstrate the factor structure and internal consistency of the Caregiver Burden Inventory (CBI) among the studied caregivers of disabled persons and to determine the effects of patients’ and caregivers’ characteristics on the burden and its dimensions.

**Methods:**

A cross-sectional study among 260 family caregivers of disabled patients was carried out in a randomly chosen rural area, Minia, Egypt, 2019. Exploratory factor analysis (EFA) was conducted to determine the factorial validity of the CBI. Multiple linear regression was used to identify the significant factors affecting the burden.

**Results:**

Factor analysis resulted in a five-factor solution using 20 items (four for each dimension) accounting for 72.7% of the total variance. The CBI and its dimensions showed high internal consistency (Cronbach’s alpha value > 0.70). Education of caregiver, family income, mental impairments, and mixed disabilities were significant predictors of total CBI burden.

**Conclusions:**

CBI is an effective multidimensional measure of the caregiver burden of disabled subjects. Caregivers experienced a distinct level of burden that is determined by caregiver and care recipient characteristics. Therefore, support and individualized counseling services should be optimized.

## Background

Disability is a dynamic, multidimensional, and diverse public health issue of increasing importance and rising trend [1]. People with a disability have been defined by The UN Convention on the Rights of Persons with Disabilities (UNCRPD) as those who have a long-term physical impairment which is the most common type, mental, intellectual or sensory impairments which in interaction with various barriers may hinder their full and effective participation in the society on an equal basis with the others [2]. Disability may be temporary due to acute disease, and/or injury, or chronic; and it may arise as a congenital condition or acquired later in life [3]. People with disabilities are one of the most disadvantaged, marginalized, and excluded population groups in terms of employment, educational attainment, access to adequate services as well as social participation and individual autonomy [4].

Disability rates are increasing due to many factors such as wars, aging population, increasing poverty and illiteracy, upward rise in chronic health conditions, and weakness of infrastructure of services [5]. Globally, people who live with some form of disability constitute approximately 15% or an estimated 1 billion people [1]. In Egypt, disability rates are reported to be very low (0.7% of the total population) [6] which are much lower than the international data-sets [7]. Thus, the figures seem unrealistic and inconclusive. This may be due to the method of data collection, starting with the understanding or definition of disability, the method of data collection, and/or societal or cultural attitudes (e.g. tending to hide disability in public). The major causes of disability in Egypt are congenital abnormalities, followed by injuries/accidents, old age, epidemics, and other diseases, and birth-related conditions [8].

Disability does not only affect the person who is disabled but also has an impact on the entire family because disabled persons require help in performing their daily activities and in managing medications [9, 10]. Parents, especially mothers, take on the responsibility as primary caregivers helping disabled individuals in maintaining their community connections [11]. The challenges faced by the caregivers are known as the “caregiver burden” which was described as a feeling of heavy responsibility, constant worries, and uncertainty about patients’ needs and constraints in caregivers’ social life [12]. As the burden increases, caregivers may be subjected to physical and mental health problems, financial burdens, disturbance in family life, and it may end with the loss of control especially if they do not receive adequate training, guidance, and support [13]. Moreover, caregivers tend to place a low priority on their own health compared to the time and effort they spend in caring for disabled members [14]. Consequently, their quality of life may be compromised especially with increasing duration, the severity of illness, and negative feelings associated with caregiving such as self-blame, guilt, shame, and embarrassment [15].

Although caregiver burden has been measured in a variety of ways [16–18], the Caregiver Burden Inventory (CBI) is a multidimensional scale aimed to assess the impact of the burden on different aspects of a caregiver’s life, including caregiver’s well-being and function which may be differentially affected by the relative’s disability. The CBI conceptualizes the burden in terms of five categories (time dependency, emotional health, physical health, development, and social relationships) [19].

Data on the prevalence of disability in Egypt are inconclusive and do not accord with international data-sets. However, the few available data suggest a high and unmet need for health-related rehabilitation [8], which may increase the burden of care for their families. Many researches have investigated the impact of disability on the persons affected, but there is a paucity of data regarding caregiver burden especially in Upper Egypt. Additionally, short term respite programs are extremely limited in rural Egypt despite the rapidly increasing demands for home services. Consequently, family members bear the heavy responsibilities and stress associated with providing appropriate care and supervision to their disabled relatives. Neglect and inadequate care are more common when the caregiver is burdened leading to deterioration in the patient’s health status which indicates and predicts the caregiver’s breaking point. Ensuring a healthy unburdened caregiver is important to secure patient safety and care effectiveness. So, this research provides an insight into this burden which may help service providers to develop new strategies to support caregivers in their role. The purpose of this study is to demonstrate the factor structure and internal consistency of the CBI to be used among caregivers of disabled persons in Minia, Egypt and to determine the associations of caregiver burden and its different dimensions with various care recipients’ and caregivers’ characteristics.

## Methods

### Study design and setting

A community-based cross-sectional study was conducted from September to November 2019 in a rural area (Nazlet El-falahin) which was randomly chosen among all 40 villages of Minia district, Egypt. The data were collected in two phases. Firstly, a list of households with disabled individuals was obtained from both the health office of the village and the non-governmental organization for the disabled support in the region. Appropriate verification of the disability state was done by contacting the households and/or the health center of the village for documentation of impairment in the form of a hospital document, physician statement, or investigation report. In case of not providing a document for a disability, the household was excluded from the list. Secondly, the study sample was recruited via a systematic random sampling technique. The first house was chosen randomly then every second house was visited in a randomly selected direction and the houses which were locked at the time of the survey were revisited on three consecutive days before excluding from the study.

### Study population and data collection

The participants were family caregivers of adolescent and adult disabled patients. Disabled persons included subjects who had been previously diagnosed to have any of the following; blindness/low vision, hearing impairment, physical disability, mental illness, and mixed impairments. The physical impairment substantially limits the person’s physical functioning, mobility, dexterity, or stamina. While a mental disability includes intellectual, speech or learning disabilities, mental or psychological disorder, and chronic neurological conditions that substantially limits one or more major life functions. Caregivers were recruited on the basis of the following criteria: a) caregivers of people aged ≥10 years with any type of disability; b) primary caregivers; as persons who adopting functions of care and supervision in basic daily activities of the disabled person c) giving care to the patient for at least 6 months; d) aged 18 years or more. Those who were paid in return for caregiving service; were excluded from this study. Among the total 260 primary caregivers eligible for the study, 17 refused to participate giving a response rate of 93.5%. Each participant was interviewed during a home visit, the aim of the study was explained and the answers to the questionnaire were filled in by the researcher.

Data collection tools included the following:

1. *A structured interview questionnaire:*

Interviews took place in the caregiver’s home. The questionnaire covered socio-demographic characteristics of both the caregiver and care recipient, the nature of the relationship with care recipients, type of disability, and the duration of caregiving/year.

2. *Caregiver burden:*

The caregiver burden was assessed using the Caregiver Burden Inventory (CBI) developed by Novak and Guest [19]. It is a 24-item multi-dimensional questionnaire measuring caregiver burden with five subscales: (a) Time Dependence; (b) Developmental; (c) Physical Burden; (d) Social Burden; (e) Emotional Burden. All dimensions contain five items with a 5-point Likert scale except Physical Burden which is based on four items. Briefly, time dependence burden evaluates stress caused by the restriction of one’s personal time due to time demands of caregiving whereas developmental burden describes a sense of failure in development with respect to their peers. Physical burden refers to the impact on caregivers’ physical health, strength, and energy while social burden implies feelings of role conflict concerning one’s job or family. Finally, emotional burden represents negative feelings, embarrassment or feeling of shame caused by the patient [19].

### Statistical analysis

Statistical analysis was carried out using IBM SPSS Statistics, version 22. Characteristics of caregivers and care recipients were expressed by mean ± standard deviation for continuous variables and as a percentage for categorical variables. In order to determine the factorial validity of the CBI among the study population, an exploratory factor analysis (EFA) was conducted using principal axis factoring. An oblique rotation using direct oblimin was implemented. Kaiser-Meyer-Olkin (KMO) measure of sampling adequacy and Bartlett’s Test of Sphericity were conducted to indicate that EFA was appropriate. The number of factors was determined according to: 1) eigenvalue > 1; 2) a scree plot; 3) the percent of variance extracted, and; 4) the interpretability of the extracted factors in the context of the research. A factor loading > 0.32 for a variable was used to be considered adequately loaded on a factor. Cronbach’s alpha coefficient was used to assess the internal consistency of the scales and alpha coefficients equal to or greater than 0.70 were considered to be satisfactory.

Bivariate associations between dimensions of burden and patients’ and caregivers’ characteristics were examined by student t-test and ANOVA. Multiple linear regression analysis was used to identify the significant factors that affect the level of caregivers’ burden. The level of statistical significance was set at *P* < 0.05.

## Results

### Sample description

The characteristics of the participants were shown in Table 1. Most of the caregivers were females (87.7%), married subjects (80.7%), and illiterates (58%). The mean age of caregivers was 43.6 ± 12.8 years and only 14% of them had a paid job. Most of the participants consist of parents (62.6%) of the disabled person, and the duration of caregiving ranged from 1 year to 40 years with a mean of 14.3 ± 9.9 years. About one third (*n* = 90; 37%) of the sample reported a worsening in their economic level. The mean age of care recipients was 31.7 ± 18.8, and 73.3% were males. Of the 243 care recipients, 141 (58%) suffered from physical disabilities.

**Table 1:** Socio-demographic characteristics of the caregivers and related care recipients

Socio-demographic characteristics	Caregivers *n* = 243	Care recipient *n* = 243
	N (%)	N (%)
Age (mean ± SD)	43.6 ± 12.8	31.7 ± 18.8
10–< 18 years	–	66 (27.2)
19–40 years	103 (42.4)	119 (49.0)
41–60 years	116 (47.4)	30 (12.3)
> 60 years	24 (9.9)	28 (11.5)
Sex		
Male	30 (12.3)	178 (73.3)
Female	213 (87.7)	65 (26.7)
Marital status		
Married	196 (80.7)	73 (30.0)
Unmarried	47 (19.3)	170 (70.0)
Working status		
Not working	209 (80)	207 (85.1)
Working	34 (14)	36 (13.9)
Education		
Illiterate	141 (58)	122 (50.2)
Primary	34 (14)	64 (26.3)
Secondary/ University	68 (28)	57 (23.5)
Relationship to the patient		
Parent	152 (62.6)	–
Spouse/partner	36 (14.8)	
Sibling	26 (10.7)	
Other relatives	29 (11.9)	
Duration of caregiving	14.3 ± 9.9	–
Type of disability		
Visual/ hearing		50 (20.6)
Physical		91 (37.4))
Mental		61 (25.1)
Mixed		41 (16.9)

### Exploratory factor analysis

The EFA results in a five-factor solution accounting for 72.7% of the total variance (Table 2).

**Table 2:** Factor Structure for the dimensions of CBI

Items of CBI	Factor 1: Physical burden	Factor 2: Time dependence burden	Factor 3: Developmental burden	Factor 4: Emotional burden	Factor 5: Social burden
Item 14: I’m physically tired	0.95				
Item 13: Caregiving has made me physically ill	0.94				
Item 12: My health has suffered	0.54				
Item 11: I’m not getting enough sleep	0.49				
Item 1: My care receiver needs my help to perform many daily tasks		0.87			
Item 4: I have to help my care receiver with many basic functions		0.81			
Item 3: I have to watch my care receiver constantly		0.67			
Item 5: I don’t have a minute’s break from my caregiving chores		0.60			
Item 9: I feel emotionally drained due to caring for my care receiver			- 0.96		
Item 7: I wish I could escape from this situation			- 0.86		
Item 6: I feel that I’m missing out on life			- 0.82		
Item 10: I expected that things would be different at this point in my life			- 0.57		
Item 21: I feel ashamed of my care receiver				0.90	
Item 20: I feel embarrassed by my care receiver’s behavior				0.87	
Item 23: I feel uncomfortable when I have friends over				0.65	
Item 24: I feel angry about my reactions toward my care receiver				0.39	
Item 16: My caregiving efforts aren’t appreciated by others in my family					0.68
Item 19: I feel resentful of other relatives who could but do not help					0.56
Item 17: I’ve had problems with my marriage					0.40
Item 15: I don’t get along with other family members as well as I used to					0.36
Explained Variance	44.8%	10.4%	6.5%	6%	5%

Unlike the original CBI which had five items in each dimension except the physical one (that had four items), and was originally supported by Caserta et al. [20] who examined the multidimensional nature of CBI, the current study showed four items with significant loadings on each retained factor. Item 2 (“My care receiver is dependent on me”), item 8 (“My social life has suffered”), item 18 (“I don’t do as good a job at work as I used to”) and item 22 (“I resent my care receiver”) were excluded during the analysis because they consistently load > 0.32 across major factors after various oblique rotations and solutions. Each extracted factor contains four items, with a scoring system ranging from 0 (minimum stress) to 4 (maximum stress). The score for each factor was calculated by multiplying the sum of items by 1.25 so that the scores for all factors can range from zero to 20 and the total score from zero to 100 to be comparable with previous studies.

The CBI scale and its dimensions proved to be very reliable (Cronbach’s alpha value for the total scale, physical, time dependence, developmental, emotional, and social burden were 0.93, 0.91, 0.84, 0.91, 0.85 and 0.77 respectively).

### Relationship of dimensions of burden and caregiver’s characteristics

The mean score of CBI for the caregivers was 48.9 ± 17.9. Among the five dimensions of burden, the time-dependent burden scored the highest (13.1 ± 5) followed by physical burden (9.9 ± 4.9). Developmental and social burden scored (9.1 ± 4.8 and 8.9 ± 4.1 respectively) while emotional burden (7.9 ± 4.2) had the lowest scores. The caregiver’s characteristics were associated with caregiver burden as shown in (Table 3). Caregivers aged > 60 years old had significantly higher physical and time dependence burden (13.4 ± 4.5 and 15.5 ± 6.5) compared to younger age groups. Non-educated caregivers had higher scores for total burden and its dimensions except the emotional burden. Moreover, low family income was associated with higher burden scores.

**Table 3:** Total and relative CBI dimension scores according to caregivers’ characteristics

Socio-demographic characteristics	CBI total	Physical burden	Time dependence burden	Developmental burden	Emotional burden	Social burden
Age groups						
< 40 years	46.4 (17.9)	9 (4.9)	12.8 (4.9)	8.9 (4.8)	7.2 (4.2)	8.3 (3.8)
41–60 years	49.7 (17.3)	10.1 (4.8)	12.7 (5.1)	9.1 (4.9)	8.4 (4.2)	9.4 (4.4)
> 60 years	55.9 (18.3)	13.4 (4.5)	15.5 (4.6)	9.3 (4.9)	8 (4.6)	9.7 (3.9)
*P-value*	0.051	<0.001	0.042	0.966	0.109	0.114
Sex						
Male	47.9 (14.7)	9.9 (5.1)	12.6 (5)	9.4 (4.2)	7.7 (3.7)	8.3 (3.8)
Female	49.1 (18.3)	10 (4.9)	13.1 (5)	9 (4.9)	7.9 (4.3)	9 (4.2)
*P-value*	0.736	0.970	0.596	0.713	0.752	0.381
Marital status						
Unmarried	48.9 (17.7)	9.9 (4.9)	13.3 (5)	9.1 (4.8)	7.8 (4.2)	8.9 (4.2)
Married	48.8 (18.9)	10.1 (5)	12.2 (5)	9.1 (4.9)	8.5 (4.4)	9 (3.9)
*P-value*	0.955	0.808	0.186	0.996	0.304	0.991
Working status						
Not working	49.2 (18.3)	10 (5)	13.1 (5.1)	9.1 (4.9)	7.9 (4.3)	9 (4.1)
Working	47.2 (14.7)	9.6 (4.5)	13 (4.9)	8.8 (4.2)	7.4 (3.6)	8.3 (4)
*P-value*	0.567	0.643	0.975	0.815	0.484	0.372
Education						
Not educated	51.5 (18.4)	10.7 (5.1)	13.6 (4.9)	9.6 (5)	8.1 (4.2)	9.5 (4.4)
Educated	44.5 (16.1)	8.6 (4.3)	12.2 (5)	8.1 (4.3)	7.6 (4.3)	7.9 (3.4)
*P-value*	0.003	0.001	0.044	0.020	0.387	0.004
Relationship to the patient						
Spouse/partner	51.5 (19.2)	10.8 (5.1)	13 (5.1)	9.3 (4.9)	8.5 (4.6)	9.8 (4.4)
Parent	44.7 (13.5)	9.4 (3.9)	13.9 (5.8)	7.4 (3.9)	6 (2.5)	7.9 (2.7)
Sibling	41.8 (8.9)	7.5 (4.5)	13.8 (4.1)	8.4 (3.2)	5.3 (0.9)	6.8 (1.9)
Other relatives	45.8 (16.2)	8.6 (4.5)	12.7 (4.8)	9.1 (4.9)	7.5 (3.9)	7.9 (3.6)
*P-value*	0.043	0.004	0.755	0.357	0.006	0.002
Duration of caregiving						
1–10 years	46.6 (16.5)	9.1 (4.7)	13.2 (4.8)	8.7 (4.4)	7.4 (4)	8.2 (3.8)
> 10 years	50.9 (18.8)	10.7 (5.1)	12.9 (5.2)	9.4 (5.2)	8.4 (4.4)	9.6 (4.3)
*P*-value	0.055	0.014	0.657	0.254	0.064	0.007
Family income						
Not/hardly enough	50.11(18.1)	10.3 (5.1)	13.3 (4.9)	9.3 (4.9)	8.1 (4.3)	9.1 (4.2)
Enough	38.4 (10.9)	7.2 (2.7)	10.8 (5.2)	6.9 (3.4)	6.1 (2.3)	7.4 (2.8)
*P-value*	<0.001	0.003	0.020	0.004	0.001	0.007
Total	48.9 (17.9)	9.9 (4.9)	13.1 (5)	9.1 (4.8)	7.9 (4.2)	8.9 (4.1)

The data was presented as mean (SD)

Spouses of patients had significantly higher scores than other related groups; (10.8 ± 5.1) for physical; (8.5 ± 4.6) for emotional; (9.8 ± 4.4) for social and (51.5 ± 19.2) for total burden. Caregiving for more than 10 years was associated with significantly higher physical and social burden scores (10.7 ± 7.1 and 9.6 ± 4.3) compared to caregiving for ≤ 10 years (9.1 ± 4.7 and 8.2 ± 3.8), respectively. The five dimensions and the total scores for CBI were not significantly associated with caregiver sex, marital status, or employment status (*P* > 0.05).

### Relationship of dimensions of burden and care recipient’s characteristics

Of the demographic factors of care recipients associated with the burden of care, caring for male patients was significantly associated with higher social burden (9.3 ± 4.2) compared to caring for females (7.9 ± 3.7). The type of disability was significantly associated with the caregiver’s total burden and its five dimensions’ scores; those who care for patients with mixed disabilities had higher scores in the total CBI and all dimensions of burden (Table 4).

**Table 4:** Total and relative CBI dimension scores according to care recipients’ characteristics

Socio-demographic characteristics	CBI total	Physical burden	Time dependence burden	Developmental burden	Emotional burden	Social burden
Age groups						
< 18 years	51.3 (18.5)	10.9 (5)	12.9 (5.1)	10 (5.1)	8 (4.3)	9.4 (4.4)
19–40 years	48.9 (18.5)	9.9 (4.9)	12.8 (5.1)	8.7 (4.8)	8.2 (4.6)	9.3 (4.2)
41–60 years	47.7 (18.7)	9.4 (5.2)	13.1 (5.2)	9.2 (4.8)	8 (3.9)	8 (3.2)
> 60 years	44.7 (11.3)	8.4 (4.3)	14.3 (4.3)	8.4 (4.1)	6 (2.2)	7.6 (3.4)
*P-value*	0.415	0.117	0.562	0.285	0.083	0.110
Sex						
Male	49.7 (18.2)	10.3 (4.9)	12.9 (5.2)	9.1 (4.9)	8 (4.3)	9.3 (4.2)
Female	46.8 (16.8)	8.9 (4.8)	13.4 (4.5)	9 (4.7)	7.6 (4.1)	7.9 (3.7)
*P-value*	0.263	0.055	0.541	0.952	0.448	0.001
Marital status						
Unmarried	49.7 (19.4)	9.8 (5.1)	13 (5.2)	9.2 (5.1)	8.6 (4.7)	9.2 (4.3)
Married	44.6 (13.3)	9.3 (4.4)	12.9 (4.9)	7.9 (3.9)	6.5 (2.7)	8 (3.3)
*P-value*	0.035	0.466	0.848	0.069	<0.001	0.043
Working status						
Not working	49.9 (18.1)	9.9 (5.1)	13.7 (4.7)	9.3 (5.2)	8 (4.4)	9 (4.1)
Working	44.4 (15.9)	9 (4.3)	11.9 (5.4)	7.8 (3.8)	7.3 (3.8)	8.3 (3.8)
*P-value*	0.032	0.188	0.016	0.033	0.263	0.249
Education						
Not educated	51.1 (17.5)	10.2 (4.8)	14.2 (4.7)	9.5 (4.9)	7.9 (4.4)	9.3 (4.3)
Educated	46.6 (18.1)	9.7 (5.1)	11.8 (5.1)	8.6 (4.7)	7.8 (4.1)	8.6 (3.9)
*P-value*	0.049	0.475	<0.001	0.191	0.815	0.190
Type of disability						
Hearing/visual	40.6 (11.2)	8.2 (3.7)	11.5 (4.6)	7.2 (3.6)	6.4 (2.3)	7.4 (2.7)
Physical	45.2 (13.7)	9.6 (4.5)	12.7 (4.8)	8.1 (3.7)	6.7 (2.9)	8.1 (3.5)
Mental	53.9 (21.5)	10.9 (5.8)	13.2 (5.3)	10.3 (5.5)	9.4 (5.2)	10.1 (4.4)
Mixed	59.8 (19.5)	11.6 (5.3)	15.5 (4.8)	11.6 (5.8)	10 (5.3)	11 (4.9)
*P value*	<0.001	0.003	0.001	<0.001	<0.001	<0.001

The data was presented as mean (SD)

### Multivariate regression for factors associated with caregiver burden

The multivariable-adjusted linear regression models (Table 5) showed that: (i) Education of caregiver, family income, mental and mixed disabilities were the significant predictors of the total CBI and developmental burden (20.3 and 18.5% of variance explained) respectively. The beta, coefficients of mental and mixed disabilities of care recipients were the highest for the total burden (11.82 and 13.74 respectively). (ii) Family income was a significant predictor of the total CBI and most dimensions, while the relationship to a patient was the only significant predictor of social burden. (iii) Having mental and mixed disabilities were the only significant predictors of emotional burden (18.5% of variance explained), while caregiver age and family income represented the significant predictors of physical burden (13.7% of variance explained). (iv) Perceived lower time-dependence burden was associated with family income and care recipient characteristics as being educated, working patients, younger age, and patients with visual or hearing impairments (15.4% of variance explained).

**Table 5:** Multivariate analyses of correlates of CBI scores

Dependent variables and significantly associated variables	Unstandardized Beta	Standardized Beta	T	*P*-value
Dependent variable: total CBI burden				
Caregiver education (educated)	−4.86	−0.14	−2.05	0.042
Family income (enough)	−12.35	−.021	−3.21	0.002
Mental disability	11.82	0.26	3.86	<0.001
Mixed disability	13.74	0.29	4.31	<0.001
Dependent variable: Physical burden				
Age of caregiver	0.098	0.29	4.22	<0.001
Family income (enough)	−3.06	−0.19	−2.76	0.006
Dependent variable: Time dependence burden				
Family income (enough)	−3.67	−0.22	−3.16	0.002
Care-recipient education (educated)	−1.32	−0.13	−1.77	0.071
Care-recipient work (working)	−3.13	−0.21	−2.87	0.005
Age of care-recipient	0.05	0.18	2.27	0.023
Hearing/visual disability	−2.56	−0.21	−2.78	0.006
Dependent variable: Developmental Burden				
Caregiver education (educated)	−1.52	−0.16	−2.24	0.022
Family income (enough)	−3.02	−0.19	−2.79	0.006
Mental disability	3.46	0.32	4.51	<0.001
Mixed disability	3.63	0.26	3.71	<0.001
Dependent variable: Emotional burden				
Mental disability	3.65	0.38	5.58	<0.001
Mixed disability	3.75	0.30	4.42	<0.001
Dependent variable: Social burden				
Relationship to the patients (being a parent)	2.13	0.27	3.78	<0.001

## Discussion

Evaluation of the burden of care of a person with a disability is an important aspect of the patient’s overall assessment. The study supports the evidence that most subjects caring for disabled patients struggled with physical, emotional, financial, and social issues [21].

### Care recipients’ and caregivers’ characteristics

The disabled subjects in this study were mainly composed of males (73.3%), 50.2% were illiterate, 13.9% were currently working, and 30% were married. These figures approximate to some extent the previous figures reported about disabled subjects in Egypt (64.2% males, 72% illiterate, 21.1% employed, and 42.4% married) [6]. Data on the prevalence of disability in Egypt are inconclusive and do not agree with international data-sets due to differences in the disability definition, data collection methods, cultural attitudes, and tendency to hide disability in public and scarce data on diseases frequently causing disabilities [8].

The study found that the caregivers were mostly women (87.7%) and non-working subjects (80%). Moreover, 58% of caregivers were illiterate. The predominance of women as caregivers corroborates the findings of previous studies [22–24] which can be explained by the tendency of women to carry out many roles, frequently including domestic work, employment, and caregiving to the family members [25]. The profiles of the caregivers in the study reveal the cultural tendency and tradition of rural communities in Egypt where participation of illiterate and non-working women in familial caregiving is preferred, especially those who have motherhood experience. Furthermore, the majority of caregivers were parents (62.6%) and spouses (14.8%). These percentages coincide with some recent findings [23, 26] and reflects the family bonding and support in the rural areas of Egypt.

### CBI as a measure of caregiver burden

The CBI questionnaire does not only quantify the global burden, but also evaluates different aspects of the burden through its subscales. The CBI has already been used in different caregiver populations [26–28] including among patients with physical, mental, and mixed disabilities [29]. This study included EFA to determine the factor structure of CBI among the study population. EFA is suitable for assessing interesting latent constructs as was intended in this study rather than to test a specific hypothesis [30]. Also, the collected data of the study was the interval level which was appropriate for EFA. Moreover, EFA accounts for measurement error and helps to result in more realistic assumptions [31] than the principal component analysis that was performed by Marvardi et al. [28]. Both the global scale and its different dimensions proved to be very reliable, as shown by the high alpha co-efficient. On the CBI scale, caregivers scored much higher on the time-dependent burden subscale compared with other subscales and the emotional burden domain showed the lowest score. These findings are consistent with the results of previous studies, which noted that the time spent on caregiving, together with the lack of sufficient personal time for the caregiver were the most important variables related to the burden of caregivers [32–34]. Similarly, Bartolo et al. [23] found that feelings of being ashamed of or embarrassed by the patient seemed to account for a smaller part of the burden.

### Determinants of caregiver burden

The study results support the finding that caregiver and care recipient characteristics may play an important role in determining the burden of care. We found that the total burden of care was associated with caregiver education and family income. Moreover, family income was associated with most subdimensions of burden. Having enough family income was associated with reduced physical, time dependence, developmental and total burden in this study. This result was consistent with previous research findings [21, 22] as higher household income is expected to lead to better living conditions and consequently a better quality of life and reduced burden of care [35].

Caregiver education affected the developmental and total burden in the study participants. A previous study showed that caregivers who are less educated, unemployed, and caring for patients for a longer duration of illness had a lower quality of life [36]. This could be attributed to the fact that better-educated caregivers would have more resources available to manage the care situation and those who had a paid job outside the house are not always available to provide full-time primary care. Caregiver age was associated with the physical burden which was supported by the previous finding which stated that older caregivers experience higher burden and greater impact on their quality of life [22, 37]. However, in contrast with Zahid and Ohaeri [38] who found that spouse caregivers reported more burden than other caregivers, we found that being a parent for a disabled person was significantly associated with increased social dimension burden.

Care-recipient characteristics including younger age, literacy, work, and having a hearing or visual impairment were associated with lower time-dependence burden. This finding was expected because disabled subjects who are educated or employed are expected to be more independent in a way that reduces the stress caused by the restriction of caregiver’s personal time.

The current study found that mental and mixed disabilities were associated with an increase in overall burden, developmental, and emotional burden. In accordance with our findings, a previous study showed that both the burden and the quality of life were significantly worse for caregivers who care for patients with both physical and mental diseases [29]. The study finding can be explained by the fact that mental and mixed disabilities include low levels of functional abilities of patients and distress due to behavioral disturbances. This means that the more physically disabled, cognitively impaired, and disturbed the patients are, the more time, emotional effects, physical and mental energy are spent taking care of them.

Other variables such as the patient’s gender and marital state, did not explain the variance of the burden of care (total and subscales) which corroborates the finding of previous studies [28]. On the other hand, statistically significant differences in caregiver burden were previously observed with the gender among spinal cord injury patients [26] and schizophrenia patients [37]. These differences in the results may be due to cultural and social factors variations between different countries that may affect the burden of care. Duration of care was not associated with the caregiver burden in the literature [21, 23] including our study as family caregivers for disabled subjects may undergo a process of psychosocial adaptation over the years, develop better care strategies, and have a less negative perception of the situation [39].

### Strengths and limitations

This study is novel in several ways including the population and the analytic methodology that we used. Most notably, previous studies about the caregiver burden of disabled persons reported the results of developed countries’ research and there is a lack of data from the Middle East region and low-income countries. Moreover, EFA suggested that only four questions were enough to investigate each dimension of CBI with the possibility to develop a reliable shorter form from CBI. However, confirmatory factor analysis should be considered in future studies to establish the construct validity of the shorter form. Furthermore, the researchers tried to minimize the interviewer bias by using the same clearly written questionnaire that were comprised of fixed-choice answers with no open-ended questions for all the study subjects. Also, keeping the questionnaire and interview language simple helped the interviewers to avoid further long explanation. The major limitation of the study is the cross-sectional design that is prone to selection bias. However, the systematic random sampling technique can help to achieve the goal of having a representative sample and reduce the possibility of bias. Additionally, persons with disabilities represent a heterogeneous group with respect to disability type and degree of severity. However, our study did not address this issue in detail which can affect the level of burden. Therefore, there is a need for future large scale studies for each type of impairment to determine the caregiver burden related to the type and severity of the disability.

## Conclusion

The study results highlight the suffering of caregivers in terms of the burden of care provided to disabled patients. CBI proved to be an effective multidimensional scale to measure the burden of care on different aspects of caregivers’ lives. Caregiver education, family income, mental impairments, and mixed disabilities were the significant predictors of total CBI burden. Support and counseling services should, therefore, be optimized, taking account of the socioeconomic conditions so that solutions tailored to caregivers’ individual circumstances are provided.

## Data Availability

The datasets used and/or analyzed during the current study are available from the corresponding author on reasonable request.

## Abbreviations

CBI: Caregiver Burden Inventory
EFA: Exploratory factor analysis
